# Slow Progression of Eruptive and Noneruptive Calcified Nodules After Atherectomy Monotherapy

**DOI:** 10.1016/j.jaccas.2026.110197

**Published:** 2026-09-11

**Authors:** Daiki Niizeki, Masataka Nakano, Tetsuya Kawamata, Kota Fukushima, Shinji Nakano, Kotaro Miyashita, Kotaro Kagiyama, Nobuhiko Ogata

**Affiliations:** a Department of Clinical Engineering, Ageo Central General Hospital, Saitama, Japan; b Department of Cardiology, Ageo Central General Hospital, Saitama, Japan; c Department of Cardiology, West Tokyo Central General Hospital, Tokyo, Japan

**Keywords:** coronary angiography, imaging, percutaneous coronary intervention

## Abstract

**Background:**

Eruptive calcified nodules (CNs) are associated with poor outcomes after conventional stent- and balloon-based percutaneous coronary intervention, leaving optimal treatment strategies uncertain.

**Case Summary:**

A 76-year-old woman presented with non–ST-segment elevation myocardial infarction. Coronary angiography and optical coherence tomography identified an eruptive CN, which was localized as the culprit lesion through functional assessment. Considering the risk of stent- and balloon-related failure, atherectomy-alone strategy, without adjunctive therapy, was performed. Serial optical coherence tomography demonstrated effective debulking with preserved vessel morphology at 5-month and 4-year follow-up without luminal compromise or recurrent events.

**Discussion:**

Given the inherently latent pathology of eruptive CN, atherectomy monotherapy, devoid of superfluous stimuli and permanent scaffolding, may be a viable strategy for selected lesions, provided sufficient ablation was obtained.

**Take-Home Message:**

A minimal atherectomy-alone strategy for the eruptive CN may yield maximal legacy, facilitating treatment options and straightforward lifetime management should recurrences occur.

## Visual Summary

**Figure:**
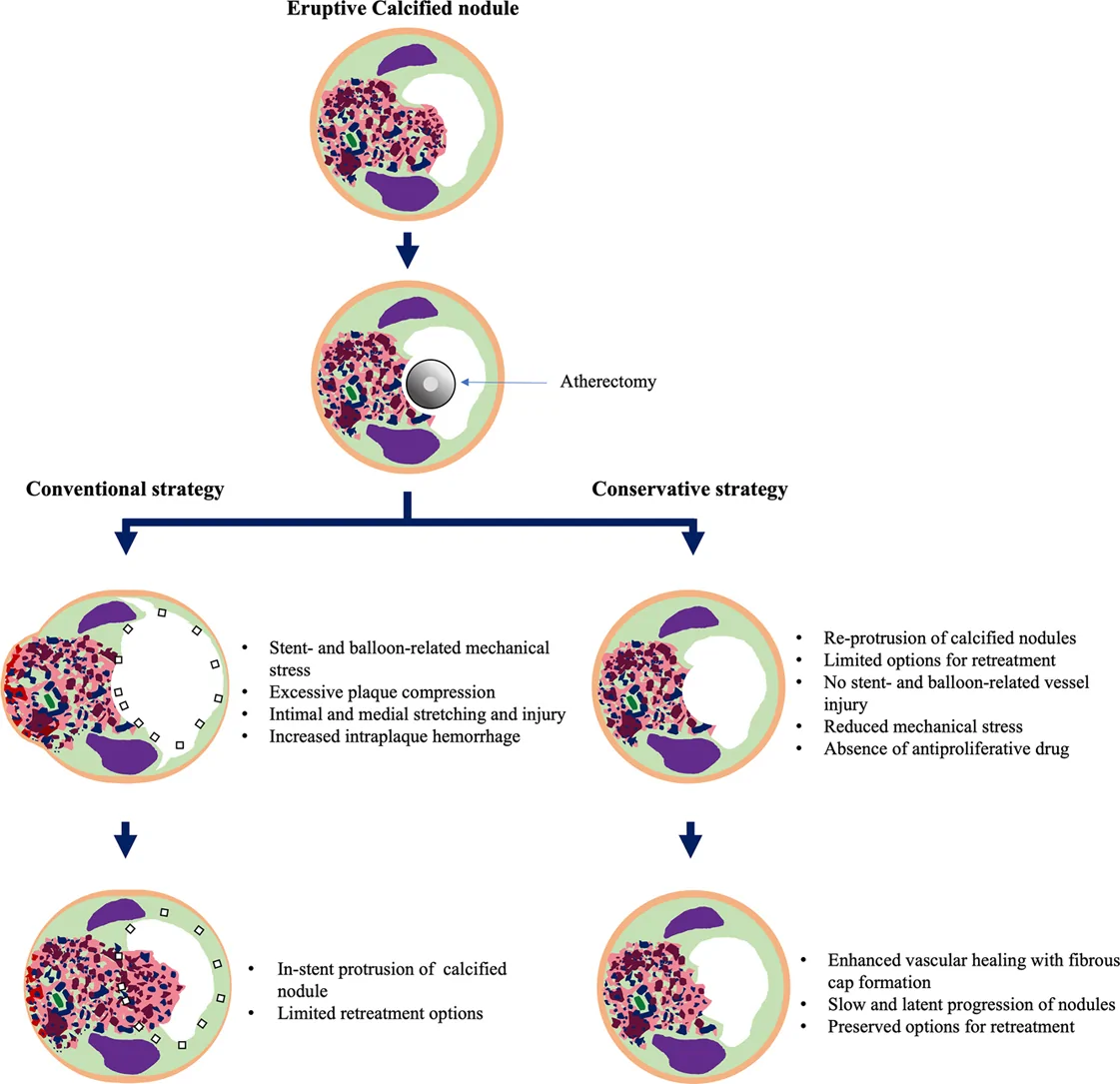

## History of Presentation

A 76-year-old woman, with a past medical history of dyslipidemia and rheumatoid arthritis, was transferred to our hospital by ambulance, presenting with nocturnal chest discomfort accompanied by nausea and vomiting. On arrival, she was hemodynamically stable, and physical examination revealed no signs of decompensated heart failure. A 12-lead electrocardiogram in the emergency room revealed ST-segment depression and T-wave inversion in leads V_3_-V_6_; however, echocardiography did not reveal any specific abnormalities such as left ventricular dysfunction or significant valvular disease. Blood tests confirmed elevated troponin I levels (0.766 ng/mL; institutional reference range, 0-0.026 ng/mL), leading to a diagnosis of non–ST-segment elevation myocardial infarction.

## Investigations

The patient was transferred to the catheterization laboratory, where coronary angiography revealed moderately stenotic calcified lesions in both the proximal left anterior descending artery (LAD) and the right coronary artery (RCA) (Figure 1). Optical coherence tomography (OCT) of the LAD and RCA revealed irregular surface protrusion with adjacent sheet calcification, consistent with the morphology of eruptive calcified nodules (CNs). Given the presence of multivessel disease, the angiographic culprit lesion could not be definitively identified, and on-site primary percutaneous coronary intervention (PCI) was deferred. On the following day, resting dual-isotope nuclear scintigraphy was performed to evaluate the ischemic territory. The results revealed early defects and delayed redistribution of thallium-201chroride (Tl), along with persistent defects of ^123^I-β-methyl-P-iodophenyl-pentadecanoic acid (BMIPP) in the anterior wall region (Figure 2). This mismatch indicated transient myocardial ischemia, confirming the LAD as the functional culprit vessel.

**Figure 1:**
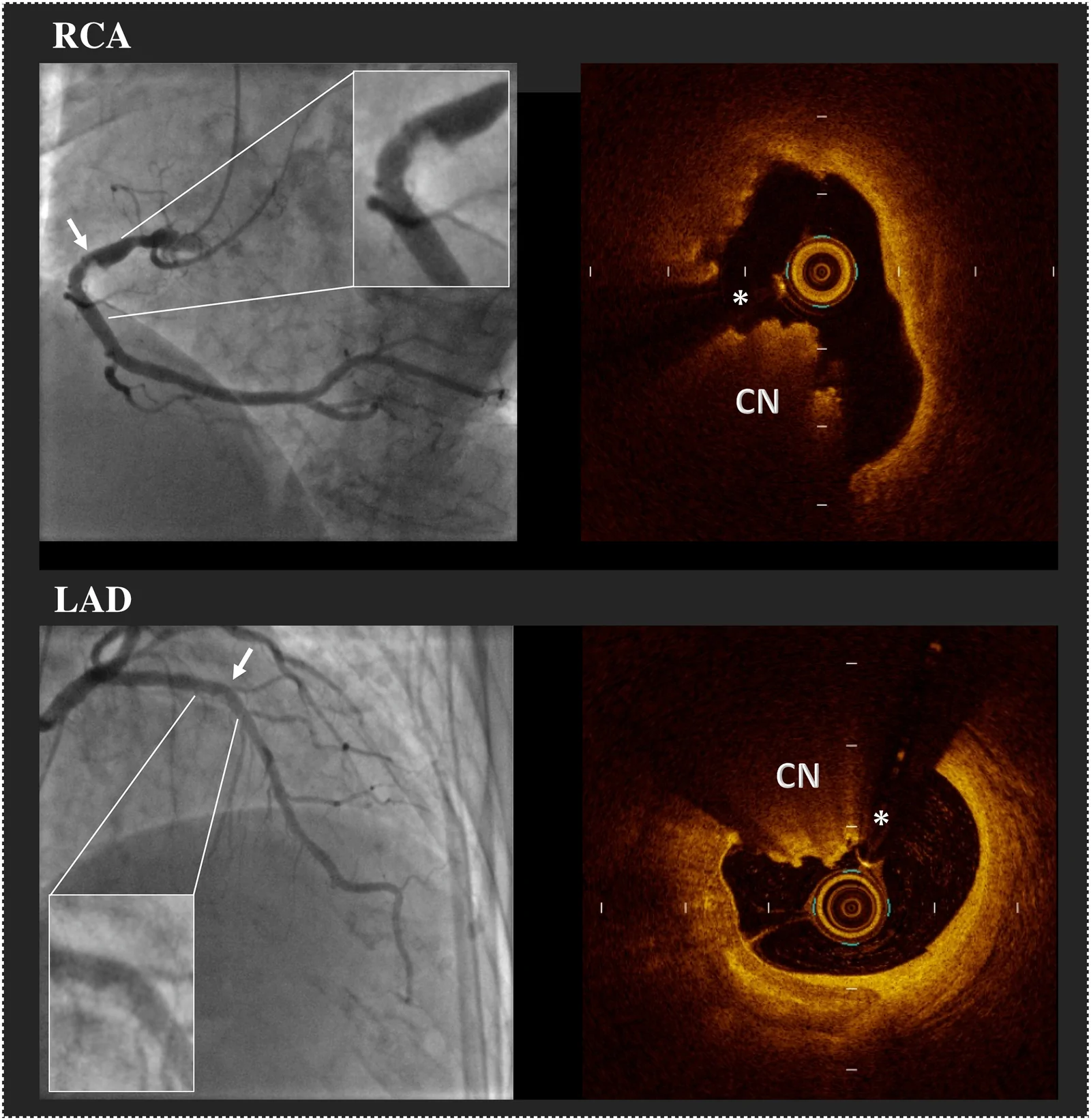
Baseline Coronary Angiography and Optical Coherence Tomography Baseline coronary angiography demonstrated moderately stenotic lesions in the proximal LAD and RCA. Corresponding optical coherence tomography revealed irregular surface morphology indicative of eruptive CN in both lesions. ∗Guidewire artifact. CN = calcified nodules; LAD = left anterior descending artery; RCA = right coronary artery.

**Figure 2:**
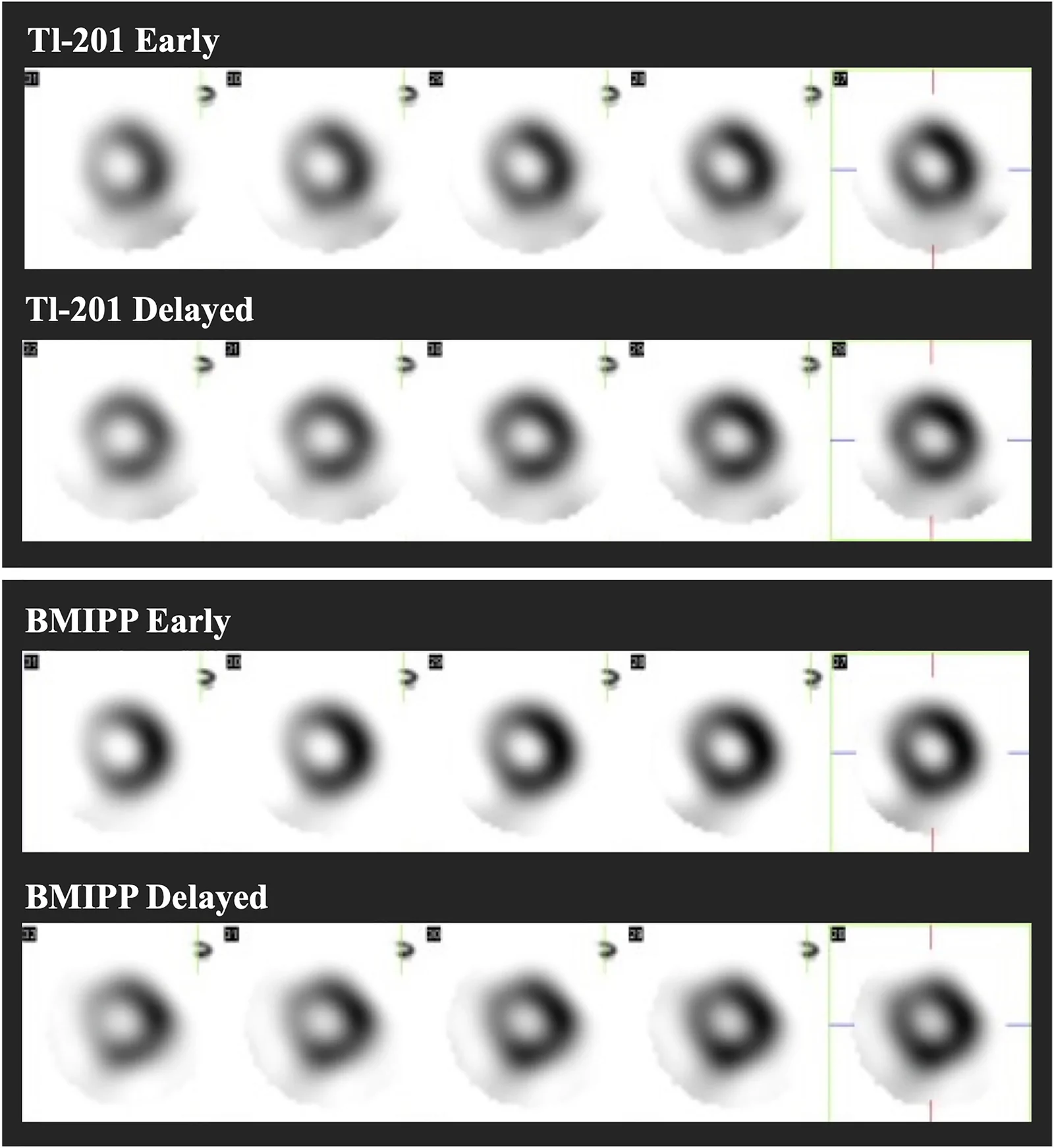
Myocardial Scintigraphy Findings The results of dual-isotope myocardial single-photon emission computed tomography revealed early defects and delayed redistribution of Tl-201 and persistent BMIPP defects in the anterior wall region, indicating transient myocardial ischemia in the left anterior descending artery territory. BMIPP = iodine-123 β-methyl-p-iodophenyl-pentadecanoic acid; Tl-201 = thallium-201.

## Management

The decision was made to proceed with PCI for the LAD lesion. Based on the OCT findings and concerns for suboptimal long-term outcomes, a stentless treatment strategy was selected. Rotational atherectomy was performed using a 2.0-mm burr, followed by a 2.15-mm burr (Figure 3). Postatherectomy OCT demonstrated adequate reduction in CN volume and sufficient lumen gain, with minimum lumen area of 6.45 mm^2^ and % area stenosis of 48%. There was no flow-limiting dissection or significant thrombus formation. No adjunctive treatments, such as plain balloon dilation, drug-coated balloon, or stent implantation, were performed. Dual antiplatelet therapy with aspirin and prasugrel was initiated during the periprocedure period and switched to aspirin monotherapy after 5 months.

**Figure 3:**
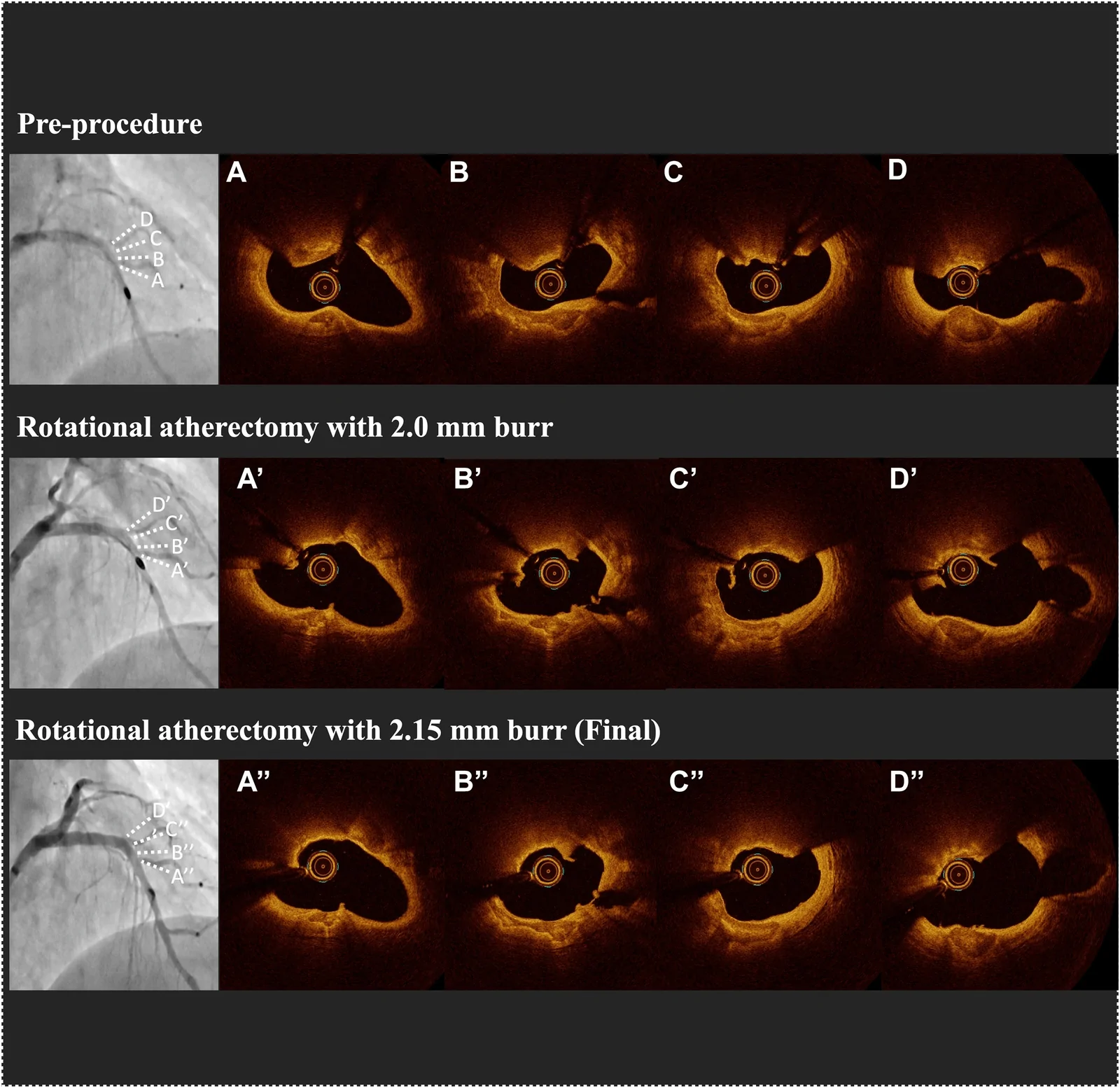
Serial Optical Coherence Tomography Changes During Rotational Atherectomy Preprocedural optical coherence tomography showing eruptive calcified nodules with luminal narrowing (A to D). After rotational atherectomy with a 2.0-mm burr, partial debulking and lumen enlargement were observed (A′ to D'). Final optical coherence tomography after additional atherectomy with a 2.15-mm burr showed further modification and improved luminal geometry (A″ to D″).

## Outcome and Follow-Up

At the 5-month follow-up, coronary angiography showed no progression of the treated LAD lesion. Intracoronary OCT images revealed no indications of thrombus formation or neointimal proliferation, which is nearly analogous to the previous luminal morphology observed at the time of the initial PCI procedure (Figure 4). Additional physiological assessment demonstrated nonischemic values (RCA: resting full-cycle ratio 0.98, fractional flow reserve 0.93; LAD: resting full-cycle ratio 0.91, fractional flow reserve 0.87).

**Figure 4:**
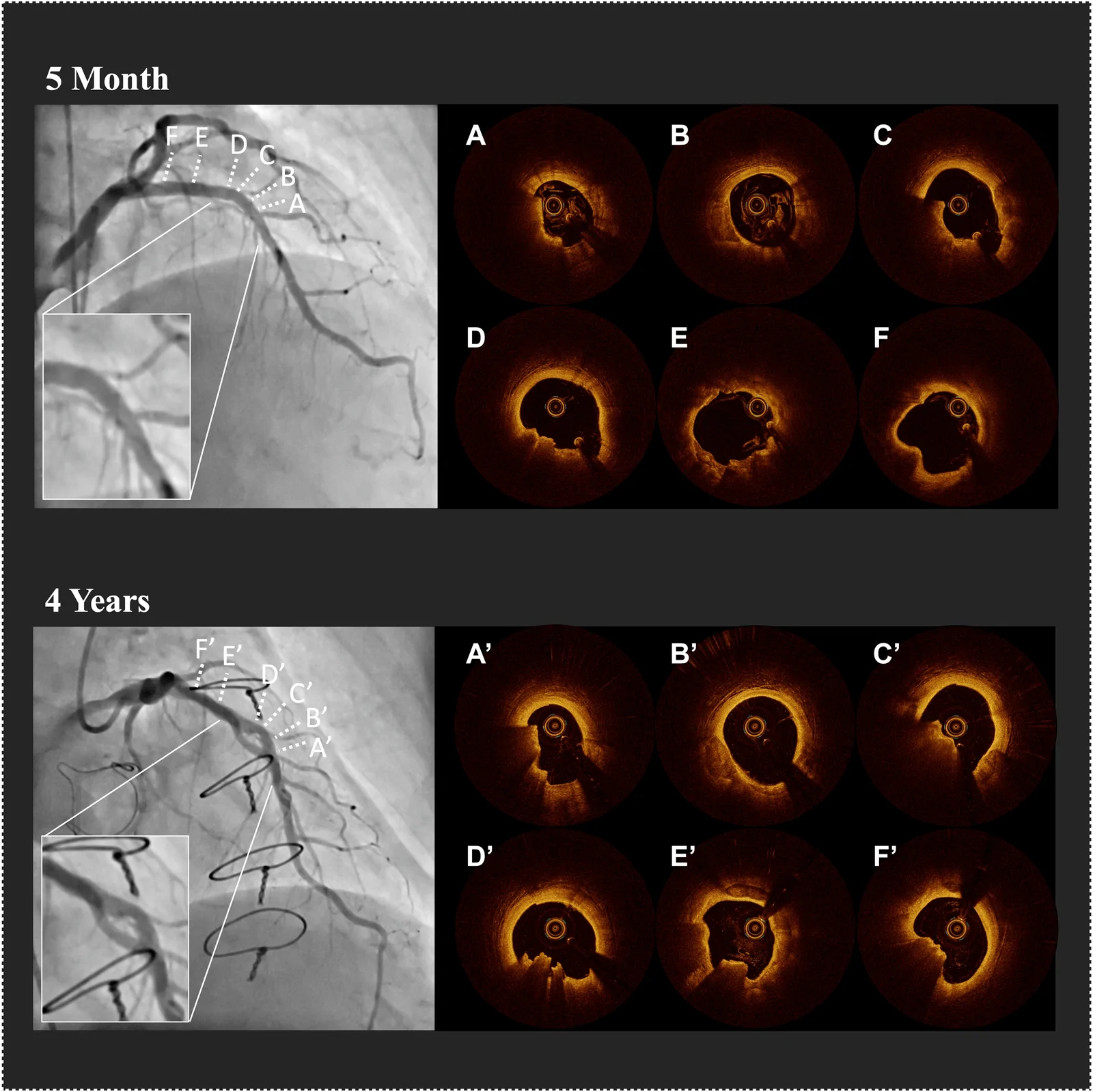
Serial Coronary Angiography and Optical Coherence Tomography at 5-Month and 4-Year Follow-Up Coronary angiography at 5 months showed no significant changes from the initial percutaneous coronary intervention (left image in upper row). Intracoronary optical coherence tomography images further confirmed the preservation of luminal morphology (right images in upper row: A to F correspond to cross-sections A to F of the angiography). At 4 years after the initial procedure, coronary angiography showed minor progression of luminal narrowing (left image in lower row). Optical coherence tomography revealed mildly increased luminal irregular protrusion, suggestive of latent progression of calcified nodules and nodular calcification, without significant luminal compromise (right images in lower row: A' to F' correspond to cross-sections A' to F' of the angiography).

At the 4-year follow-up, coronary angiography confirmed sustained luminal patency. OCT demonstrated, for the most part, unchanged luminal morphology, although mild progression of remnant eruptive CNs and transition from noneruptive intraplaque nodular calcific components to slight eruption into the lumen were observed at the atherectomy-treated site without significant luminal compromise (Figures 4 and 5). The patient remained asymptomatic on aspirin monotherapy.

**Figure 5:**
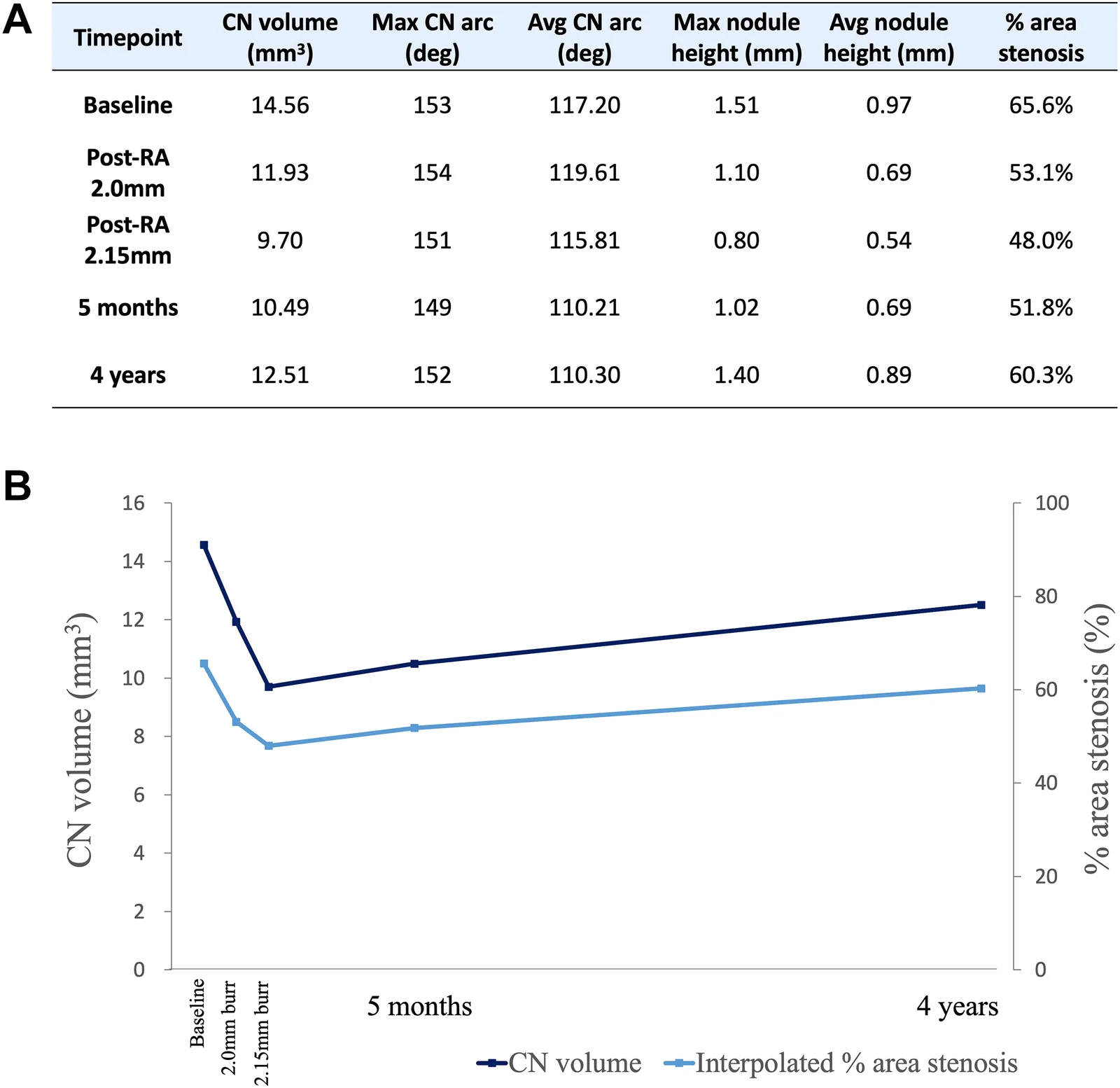
Quantitative Analysis of Calcified Nodule Morphology and Luminal Narrowing Over Time (A) Serial changes in optical coherence tomography measurements of CN volume, arc, and height, with interpolated percent area stenosis. (B) Temporal trends showing an initial reduction after atherectomy followed by minimal increase, consistent with slow progression. CN = calcified nodules; RA = rotational atherectomy.

## Discussion

Among the various pathological substrates precipitating acute coronary syndrome, a CN is of particular significance as it is associated with a poorer prognosis than plaque rupture or erosion.^1^ The precise mechanism underlying the formation of CNs remains unclear; however, cross-sectional microscopic examination suggests that fragmentation of sheet-like calcifications, as well as intraplaque hemorrhage and degeneration, play an important role in the early phases of nodular calcification formation (noneruptive CN). In later phases, it is presumed that disruption of the fibrous cap consequently results in the formation of protruding thrombogenic milieu, which is referred to as “eruptive CN.”^2^

PCI for eruptive and noneruptive CNs is reported to be challenging. In particular, treatment with stent implantation for eruptive CNs demonstrated a higher rate of target lesion revascularization at 2 years compared with noneruptive CN (18.3% vs 9.6%, *P* = 0.048), despite achieving better stent expansion.^3^ These findings suggest that favorable acute angiographic results may not translate into durable clinical benefit in eruptive CN lesions treated with permanent scaffoldings.

The explanations for unfavorable outcomes of stenting for CN have already been documented through histopathological and clinical imaging studies: Individual tiny nodules traverse the interstrut void, subsequently protruding back into the in-stent space. Alternatively, vessel injury can induce fibrous cap disruption, resulting in intraplaque nodular calcifications protruding into the lumen.^4^^,^^5^ In certain instances, this characteristic of CN can result in a detrimental cycle wherein frequent retreatment is required following stent placement.^6^ Presumably, for the same reason, the effectiveness of drug-coated balloons is also limited.^7^^,^^8^ In the present case, given the aforementioned considerations, a strategy of atherectomy monotherapy was used, without adjunctive treatment such as plain ballooning or drug-coated balloon, because of the following concerns: 1) the outward force exerted by the stent to displace the CN could actually augment the rebound force; 2) it could lead to intraplaque hemorrhage, leading to an enlargement of the CN; and 3) there is a possibility that inappropriate stimulation of the vessel could cause vessel injury. Atherectomy alone, in this context, may modify luminal geometry and induce less mechanical stimuli without leaving a permanent scaffold. Nevertheless, there is a scope for discussion concerning the selection of lesions that are appropriate for this strategy and the geometric targets. The absence of flow-limiting dissection or significant thrombus formation is a prerequisite for this procedure; adequate reduction of CN volume and sufficient lumen gain are also paramount. Conventional criteria such as minimum lumen area of >6.0 mm^2^ and % area stenosis of <50% may be applicable in the interim. Furthermore, reduction in the severity of CN obstruction and physiological assessments may also serve as indicators. However, further large-scale studies are necessary to establish specific indicators.

The progression of human atherosclerosis is regarded as a protracted process by nature, often taking several years before it becomes clinically manifest.^9^ In particular, the progression of calcified lesions is thought to be gradual.^10^ Indeed, the PCI treatment strategy used in this case has yielded notable outcomes, successfully stabilizing the intravascular condition through the removal of only the clinically relevant segments, thus avoiding unnecessary artificial modifications and materials to the vessel. This strategy has effectively enabled the patient to maintain an uncomplicated state for an extended period. The favorable long-term angiographic, physiological, and OCT findings in this case indicated that, in contrast to lipidic lesions, as calcified lesions generally exhibit lower thrombogenicity, they can be managed with minimal intervention and medication while undergoing close observation, provided the lumen and blood flow are maintained. However, this approach should be considered hypothesis-generating, as it is based on a single case and requires further validation in larger studies.

## Conclusions

In selected patients with clinical manifestations attributable to eruptive CN, minimal vessel modification with atherectomy monotherapy, without balloon dilation or stent implantation, may represent a viable treatment strategy. Certainly, meticulous evaluation and long-term follow-up are imperative.

**Visual Summary:**
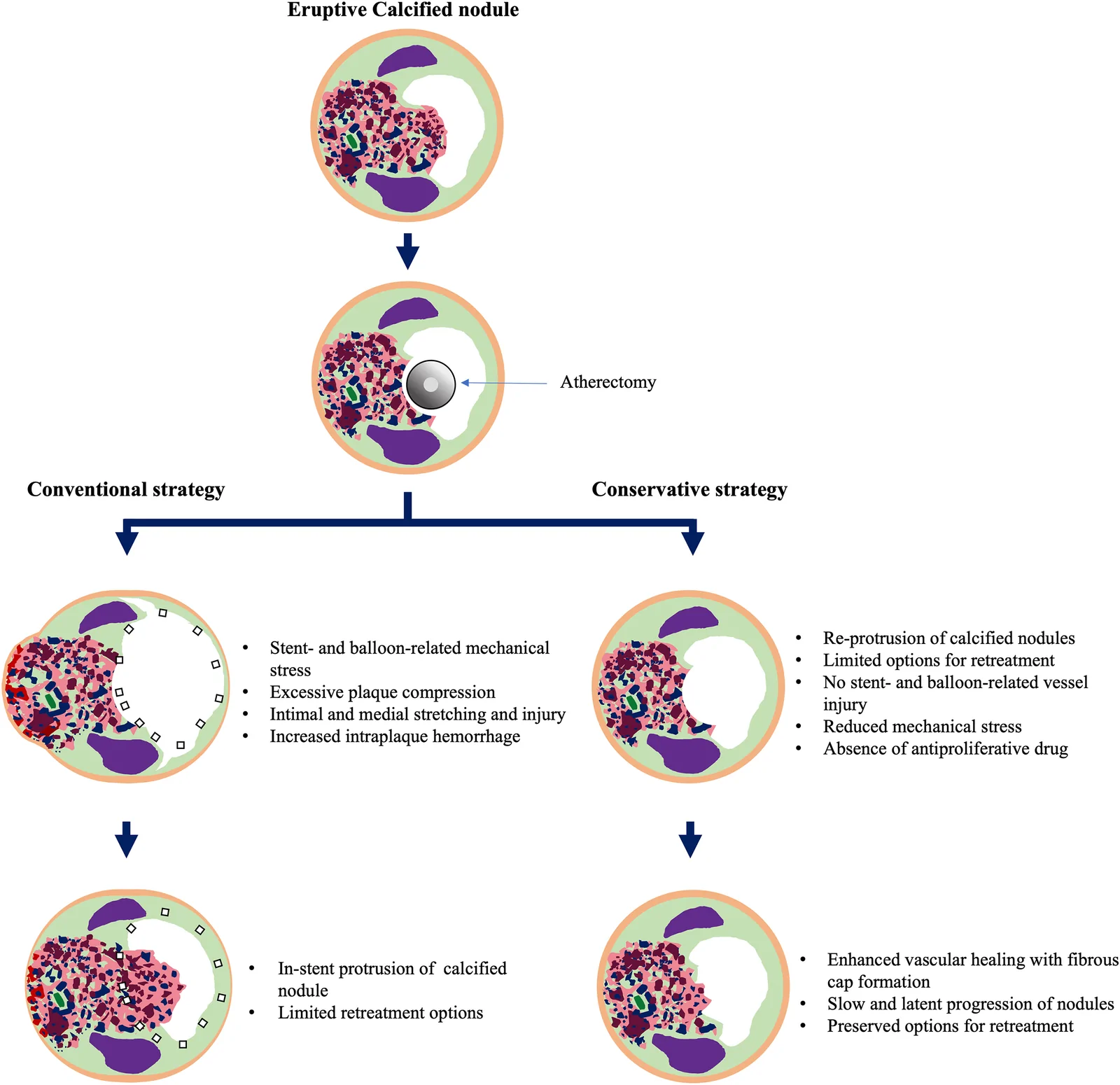
Pathophysiological Rationale for Atherectomy-Alone Strategy of Eruptive Calcified Nodules Conventional intervention involving ballooning and stenting exerts excessive mechanical stress, which may augment the rebound force or cause vessel injury and fibrous cap disruption, resulting in eruption of intraplaque nodular calcifications. Atherectomy monotherapy selectively debulks protruding components while minimizing detrimental stimulation or vessel injury and avoiding permanent scaffolding.

### Ethics Statement

This investigation was approved by the ethics committee of Ageo Central General Hospital (Saitama, Japan; approval code: 679) according to the World Medical Association Declaration of Helsinki.

**Take-Home Messages**

• Eruptive calcified nodules represent a distinct pathological entity in which conventional stent-based or balloon-based strategies may not be optimal.
• Given the underlying pathophysiology of calcified nodules, atherectomy monotherapy, aimed at minimizing vessel stimuli, could constitute a viable treatment option in cases in which the lumen area and blood flow are adequately restored.

## Funding Support and Author Disclosures

The authors have reported that they have no relationships relevant to the contents of this paper to disclose.
